# Overcoming internal challenges and external threats to noncommunicable disease control

**DOI:** 10.2471/BLT.18.228809

**Published:** 2019-02-01

**Authors:** Viroj Tangcharoensathien, Orana Chandrasiri, Orratai Waleewong, Nattadhanai Rajatanavin

**Affiliations:** a International Health Policy Program, Ministry of Public Health, Tiwanon Road, Muang District, Nonthaburi 11000, Thailand.

Despite global commitments, progress on noncommunicable disease prevention and control has been slow and uneven, particularly in low- and middle-income countries.^1^ This theme issue on noncommunicable diseases presents a selection of papers that analyse how the burden of noncommunicable diseases could be addressed more efficiently.

The World Health Organization (WHO) has recommended 16 best-buy interventions^2^ that are highly cost–effective, affordable, and could save a total of 9.6 million global premature deaths from noncommunicable diseases by 2025.^1^ Furthermore, every United States dollar (US $) invested in these interventions would yield a return of at least US $ 7 by 2030 from increased employment, improved productivity and longer life.^2^ However, the implementation status of these interventions in seven Asian countries shows that progress is uneven and capacity gaps exist.^3^ In these countries, the absence or inadequate capacity of national noncommunicable disease coordinating agencies – that is, agencies responsible for multisectoral actions to prevent and control such diseases – is a major barrier to the successful implementation of the noncommunicable disease agenda.

Papers in this issue also show that addressing risk factors is an important and cost–effective strategy, but political actions are needed to reduce exposure to these risk factors. For example, action to reduce air pollution and to mitigate climate change can address leading risk factors for noncommunicable diseases.^4^ Evidence on the effect of legislation designed to reduce unhealthy diets is also needed. A modelling paper by Saxena et al. shows that the introduction of the sweetened beverage tax in the Philippines in 2017 would not only raise government revenue, but also reduce health-care costs of noncommunicable diseases, prevent catastrophic health expenditure by patients and reduce deaths from such diseases.^5^ Onagan et al. describe the strategies employed when arguing for the introduction of this tax.^6^

To enhance the promotion of physical activity, which has multiple positive impacts on health, Rutter et al. propose a system mapping of factors contributing to activity.^7^

Health promotion campaigns are another prevention strategy. To finance such campaigns, 20 countries have used innovative financing or created health promotion foundations.^8^

External threats, combined with weak national capacities, allow industries to have a significant influence on noncommunicable disease control, for instance through threatening governments with law suits and sponsoring front groups^9^ to challenge scientific evidence on the health consequences of harmful products. Food industries employ similar tactics to those of the tobacco and alcohol industries to undermine public health policies on noncommunicable disease control, as highlighted in a paper on palm oil by Kadandale et al.^10^ Tobacco company lawyers helped to conceal evidence on the dangers of smoking and used threatening litigation that prevented successful lawsuits against their client companies.^11^ For instance, the tobacco industry has filed lawsuits against Thailand for increasing the size of health warnings and against Australia for introducing plain packaging of tobacco products. More than a decade into the implementation of the Framework Convention on Tobacco Control, State Parties have yet to fully fulfil their obligations. Magnusson et al. argues that legislative and regulatory actions are central to successful noncommunicable disease prevention and control.^12^ Legally binding instruments at international and national levels have contributed to progress on tobacco control, but have yet to be applied to the protracted challenges of the harmful use of alcohol. Furthermore, trade and investment agreements might influence the availability of commodities affecting noncommunicable disease burden. However, further studies are required to investigate the effect of such agreements at national level.^13^

Other strategies to tackle the noncommunicable disease burden are also needed, such as innovative financing mechanisms and the use of new technology and data.^14^ Countries will need to adapt their health systems to the increasing burden of noncommunicable diseases, and sharing of experience between countries will be important for implementation of new guidelines. The strategies and lessons learnt from adapting and implementing Botswana’s first primary health-care guidelines for adults are presented in this theme issue.^15^

Many countries are unlikely to achieve the global noncommunicable disease targets by 2025 unless bolder political actions are taken to translate political pledges into financial commitments. Effective implementation to address the countries’ internal challenges by strengthening national systems to better monitor progress and hold all stakeholders accountable are needed. Good governance, transparency, civil society engagement, and policy coherence across sectors would also contribute to improve countries’ capacities to address external threats and protect people’s health.
